# Increased capsaicin receptor TRPV1-expressing sensory fibres in irritable bowel syndrome and their correlation with abdominal pain

**DOI:** 10.1136/gut.2007.138982

**Published:** 2008-02-04

**Authors:** A Akbar, Y Yiangou, P Facer, J R F Walters, P Anand, S Ghosh

**Affiliations:** 1 Department of Gastroenterology, Imperial College London, UK; 2 Department of Clinical Neuroscience, Imperial College London, UK

## Abstract

**Objective:**

The capsaicin receptor TRPV1 (transient receptor potential vanilloid type-1) may play an important role in visceral pain and hypersensitivity states. In irritable bowel syndrome (IBS), abdominal pain is a common and distressing symptom where the pathophysiology is still not clearly defined. TRPV1-immunoreactive nerve fibres were investigated in colonic biopsies from patients with IBS, and this was related to abdominal pain.

**Methods:**

Rectosigmoid biopsies were collected from 23 IBS patients fulfilling Rome II criteria, and from 22 controls. Abdominal pain scores were recorded using a validated questionnaire. TRPV1-, substance P- and neuronal marker protein gene product (PGP) 9.5-expressing nerve fibres, mast cells (c-kit) and lymphocytes (CD3 and CD4) were quantified, following immunohistochemistry with specific antibodies. The biopsy findings were related to the abdominal pain scores.

**Results:**

A significant 3.5-fold increase in median numbers of TRPV1-immunoreactive fibres was found in biopsies from IBS patients compared with controls (p<0.0001). Substance P-immunoreactive fibres (p = 0.01), total nerve fibres (PGP9.5) (p = 0.002), mast cells (c-kit) (p = 0.02) and lymphocytes (CD3) (p = 0.03) were also significantly increased in the IBS group. In multivariate regression analysis, only TRPV1-immuno-reactive fibres (p = 0.005) and mast cells (p = 0.008) were significantly related to the abdominal pain score.

**Conclusions:**

Increased TRPV1 nerve fibres are observed in IBS, together with a low-grade inflammatory response. The increased TRPV1 nerve fibres may contribute to visceral hypersensitivity and pain in IBS, and provide a novel therapeutic target.

Irritable bowel syndrome (IBS) is the most common disorder presenting to gastroenterologists, with a prevalence of up to 20% in the UK and the USA.^1^ ^2^ Patients commonly present with abdominal pain associated with altered bowel habit. Self-reported abdominal pain is a very common symptom in the population including healthy individuals, but pain is more severe and frequent in patients with IBS.^3^ Untreated pain leads to a decrease in daily function capability, social stresses, loss of work and poor quality of life.^4^ Furthermore, a study carried out by Sandler *et al*^5^ revealed that abdominal pain was the symptom most likely to result in medical consultation in IBS patients. Therapeutic options currently available are limited, and often disappointing in efficacy. Functional bowel disorders such as IBS are characterised by visceral hypersensitivity,^6^ which may manifest as pain associated with bowel disturbances.^7^ Although the pathogenesis of visceral hypersensitivity is not fully understood, several mechanisms have been proposed, including subtle inflammation, psychosocial factors and altered sensorimotor function of the gut, a major component of which is believed to be peripheral and central sensitisation of visceral afferent neuronal pathways.^8^

The molecular and cellular mechanisms of the pathophysiology of IBS are increasingly the subject of study. The transient receptor potential vanilloid type-1 (TRPV1 or VR1) has been shown to play a role in animal models of inflammatory hyperalgesia. TRPV1 is expressed by sensory neurons and activated by capsaicin,^9^ heat (>43°C), acid (pH<5.9) and inflammatory mediators, with depolarisation leading to burning pain. TRPV1 activation also leads to local release of sensory neuropeptides including calcitonin gene-related peptide (CGRP) and substance P (SP) which, in turn, activate their effector cell receptors and contribute to the process of neurogenic inflammation. TRPV1 is expressed throughout the gastrointestinal (GI) tract in myenteric ganglia, muscular layers and mucosa.^10^ Our previous studies have shown changes of TRPV1 in upper and lower GI disorders: in inflammatory bowel disease, we reported greatly increased TRPV1 immunoreactivity in biopsies taken from patients with active painful Crohn’s disease compared with controls (Yiangou *et al*^10^). Our studies of biopsies from patients with Hirschprung disease^11^ and patients with idiopathic rectal hypersensitivity with faecal urgency^12^ also revealed increased TRPV1-expressing nerve fibres. In the latter study, increased levels of TRPV1-expressing nerve fibres were correlated significantly with hypersensitivity to rectal distension and mid-rectal heat stimulation (Chan *et al*^12^).

Studies which specifically address any involvement of TRPV1 in IBS are lacking. We have therefore investigated the presence of TRPV1 nerve fibres in colonic biopsies of IBS patients and controls, and related these to the degree of abdominal pain. IBS and control subjects were further characterised by parameters known to be related to IBS such as the neuropeptide SP, markers of inflammation including c-kit (for mast cells) and psychological assessments using validated questionnaires.

## MATERIALS AND METHODS

### Patients

The study was approved by Hammersmith Hospitals Research Ethics Committee and all subjects gave fully informed consent before taking part. Twenty-three unselected patients with IBS and 22 controls participated in the study (see table 1). IBS patients were undergoing either a flexible sigmoidoscopy (n = 7) or a colonoscopy (n = 16) at Hammersmith Hospital, London. IBS was diagnosed according to the Rome II criteria and the subjects were further subclassified according to Rome II criteria into either diarrhoea-predominant (IBS-D), constipation-predominant (IBS-C) or IBS with alternating stool pattern (IBS-A) (see table 2). Controls were selected from patients who were undergoing colonoscopy for other indications (such as polyp and cancer surveillance) and had a normal colon (see table 3). None of the patients was taking anti-inflammatory drugs or immunosuppressants. IBS patients had previously been seen in clinic, and other GI diseases had been excluded. Coeliac disease was excluded in all IBS patients by checking coeliac serology (immunoglobulin A (IgA), antiendomysial and antitissue transglutaminase (TTG) antibodies). Patients under active psychiatric care were excluded.

**Table 1:** Patient demographics

	Number	Age (years)	Gender (M:F)
Control	22	64 (55–75)	7:15
IBS	23	53 (30–65)	3:20
IBS-D	8	34 (26–57)	1:7
IBS-C	8	59 (49–69)	2:6
IBS-A	7	65 (24–70)	0:7

Values are the median and interquartile range.

F, female; IBS, irritable bowel syndrome; IBS-A, IBS with an alternating stool pattern; IBS-C constipation-predominant IBS; IBS-D diarrhoea-predominant IBS; M, male.

**Table 2:** Symptoms reported by patients with irritable bowel syndrome

IBS-type	Stool frequency	Stool consistency/type (Bristol stool form scale)	Frequency of abdominal pain in days/week
IBS-D	3–4/day	7	7
IBS-A	Variable	2, 3, 7	7
IBS-C	Once every 2–3 days	2, 3	6
IBS-A	Variable	3, 6	2
IBS-A	Variable	3, 6	7
IBS-A	Variable	2, 3, 6	7
IBS-D	2–3/day	7	7
IBS-C	Once every 1–2 days	3	2
IBS-C	Once every 2 days	2	1
IBS-D	3–4/day	6, 7	7
IBS-A	Variable	2, 6	2
IBS-C	Once every 1–2 days	2	0 (pain every few weeks)
IBS-D	3/day	7	6
IBS-D	2/day	6	3
IBS-C	Once every 2 days	2	3
IBS-C	Once every 1–2 days	2	1
IBS-D	4–5/day	7	7
IBS-A	Variable	2, 6	7
IBS-D	4/day	6	2
IBS-A	Variable	2, 7	5
IBS-A	Variable	3, 6	2
IBS-C	Once every 1–3 days	2	3
IBS-D	4/day	6	2

IBS-A, irritable bowel syndrome with an alternating stool pattern; IBS-C constipation-predominant irritable bowel syndrome; IBS-D diarrhoea-predominant irritable bowel syndrome.

**Table 3:** Indications for colonoscopy in the control group

Indication for colonoscopy	n
Polyp follow-up	8
Polyp follow-up; family history of colorectal cancer	1
Iron deficiency	4
Per rectal bleeding	7
Family history of colorectal cancer	2

Patients who were undergoing colonoscopy received standard bowel preparation in the form of 4 litres of Kleanprep. For flexible sigmoidoscopy patients received a phosphate enema 1 h prior to the procedure. All patients had macroscopically normal bowel mucosa on examination. A total of four mucosal biopsy specimens were taken using standard biopsy forceps from all subjects from the rectosigmoid junction in order to standardise the site of sampling. Of these, two biopsies were sent for routine H&E histological analysis to exclude any evidence of inflammation and two for immunohistochemical staining using specific antibodies.

### Questionnaires

#### Pain severity, depression and anxiety

Patients and controls were given a pain diary to complete for the 7 days prior to commencing their bowel preparation for endoscopy. The validated Short Form McGill Pain Questionnaire (SF-MPQ) was used.^13^ This has a 0–10 cm visual analogue scale for patients to record pain severity or “present pain intensity-visual analogue scale” (PPI-VAS), and a descriptive table to characterise the type of pain and formulate a sensory and affective pain score. The pain scores from these diaries were collected and the maximum recorded PPI-VAS over the 7 days, or VASmax, noted for each subject. As typically IBS patients experience episodic pain which lasts 1–2 days, the VASmax was thought to be more representative of the impact of pain on the IBS patients than the mean pain score. The mean pain score, or VASav, was also calculated.

Depression was measured using the Beck Depression Inventory (BDI).^14^ This consists of a 21-item measure with a 4-point intensity scale widely used as a self-report measure that evaluates the presence and severity of depressive symptoms. Participants were asked to complete the BDI based on how they had been feeling over the past 2 weeks. A score of >13 indicates a degree of depression.

The Hospital Anxiety and Depression Scale (HADS),^15^ which is a 14-item self-report questionnaire with a 4-point intensity scale for each item to measure anxiety and depression severity in a medical context, was given to each subject to complete. This has two subscales (anxiety and depression) with each subscale giving maximum scores of 21, and total scores range from 0 to 42. A score of 8 or above on a subscale signifies a degree of anxiety/depression.

Patients were asked to complete the questionnaires at home so they had time to comprehend and complete them and then return them via the post.

### Immunohistochemistry and histology

The two rectosigmoid biopsies were fixed in buffered 10% formalin and processed routinely for H&E histology. The histological sections were all evaluated by an experienced GI pathologist.

A further two biopsies were used for immunohistochemistry using c-kit/CD117, CD3 and CD4 antibodies to exclude inflammation or other pathology, and antibodies for TRPV1, SP and the pan-neuronal structural nerve marker protein gene product (PGP) 9.5. The primary antibodies used in the study are listed in table 4, and they were used as described previously.^12^ Tissues were snap-frozen and stored at −70°C or immersed in fixative (4% (w/v) paraformaldehyde in phosphate-buffered saline (PBS: 0.1 M phosphate; 0.9% (w/v) saline; pH 7.3)) then washed in PBS containing 15% (w/v) sucrose and 0.05% (w/v) azide for 1 h before snap freezing in embedding medium (Tissue-Tek OCT compound, Sakura Finetek, Torrance, California, USA). Frozen tissue sections (15 µm) were collected onto poly-l-lysine-coated (Sigma, Poole, Dorset, UK) glass slides. Unfixed tissue sections were postfixed in 4% (w/v) paraformaldehyde, whilst tissue sections from immersion fixed biopsies were allowed to dry on the slide (for SP and PGP9.5 antibodies only). Endogenous peroxidase was blocked by incubation in 0.3% (w/v) hydrogen peroxide in industrial methylated spirit. After rehydration in PBS, sections were incubated overnight with primary antibodies (table 4). Controls included omission of primary antibodies, or their replacement with preimmune serum. The specificity of TRPV1 immunostaining was confirmed by preincubation of primary antibodies with cognate peptide antigen as previously described by us in human intestine.^12^ Sites of antibody attachment were revealed using the nickel-enhanced, immunoperoxidase method (avidin–biotin complex, ABC elite; Vector Laboratories, High Wycombe, Bucks, UK). Nuclei were counterstained with 0.1% (w/v) aqueous neutral red.

**Table 4:** Primary antibodies

Antibody	Host	Source: Reference	Titre
TRPV1	Rabbit	GSK/C22	1:10 000
Substance P	Rabbit	Chemicon UK	1:8000
PGP9.5	Rabbit	Ultraclone	1/80 000
CD3	Mouse	Dako Cytomation, Ely, UK Clone UCHTI	1:5000
CD4	Mouse	Dako Cytomation, Ely, UK Clone MT310	1:5000
c-kit	Mouse	Novacastra, Newcastle upon Tyne, UK, clone 57A5D8	1:2000

PGP, protein gene product; TRPV1, transient receptor potential vanilloid type-1.

### Analysis of immunoreactive fibres

Immunoreactive nerve fibres and cell markers were quantified by computerised image analysis (Olympus Analysis Five DP Soft, UK). Analogue images were captured via video link to an Olympus BX50 microscope and converted into digital monochrome images by the computer. The grey-shade detection threshold was set at a constant level to allow detection of positive immunostaining, and the area of highlighted immunoreactivity in the mucosa was obtained as a percentage (% area) of the field scanned. Five fields (×40 objective magnification) per tissue section, chosen at random, were scanned, and the mean values of readings obtained by two independent blinded observers were used for final analysis. For TRPV1 analysis, as the immunostaining revealed fine fibres, the total numbers of fibres were counted per section and results were expressed as mean number of fibres/mm^2^. Further details of methods are provided in our previous paper (Chan *et al*).^12^

### Statistical analysis

Data were compared using the Mann–Whitney U test with Winstat for EXCEL software. p Values <0.05 were considered as statistically significant. Correlations between two parameters were performed using Spearman rank correlation. Univariate and multivariate linear regression models were used to assess the associations between primary antibody variables and VASmax. All data are reported as median values and interquartile range unless otherwise stated.

## RESULTS

All biopsies were reported as normal for H&E histology.

### Neuronal markers

**Figure 1:**
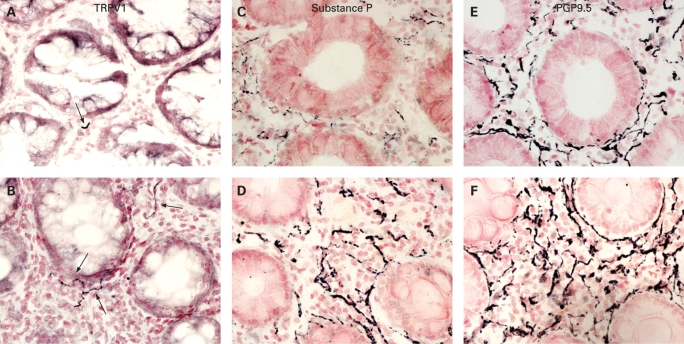
Photomicrographs showing transient receptor potential vanilloid type-1 (TRPV1)- (arrowed), substance P and protein gene product (PGP) 9.5-immunoreactive fibre immunostaining in a control (A, C and E) and an irritable bowel syndrome rectosigmoid biopsy (B, D and F), magnification ×40.

TRPV1-immunoreactive fine fibres were seen scattered throughout the mucosa in all biopsies, but were more abundant in those from IBS patients than controls (fig 1A,B). Quantitation revealed that the median number of TRPV1 fibres was significantly (3.5-fold) higher in IBS patients compared with controls (p<0.0001, table 5). SP-immunoreactive fibres (fig 1C,D) were also significantly (3-fold) greater in IBS patients compared with controls (p = 0.01, table 5), as were PGP9.5-immunoreactive fibres (p = 0.002, fig 1E,F, table 5).

**Table 5:** Quantitation of immunoreactivities in colonic biopsies from IBS and controls

	n	TRPV1	SP	PGP9.5	c-kit	CD3	CD4
IBS	23	3.2 (2.2–5.1)	2.3 (0.7–3.1)	5.0 (3.2–6.1)	4.0 (2.9–5.6)	8.7 (5.9–12.1)	1.0 (0.5–3.8)
Control	22	0.9 (0.5–2.1)	0.8 (0.5–1.5)	2.7 (1.7–3.8)	2.9 (1.9–3.9)	5.4 (3.9–7.9)	1.0 (0.3–1.8)
Ratio of medians IBS:control	–	3.56	2.96	1.86	1.36	1.60	0.98
p Value	–	<0.0001	0.01	0.002	0.02	0.03	0.29

Values are medians with interquartile range in parentheses. TRPV1 is expressed as fibres/mm^2^, and the other immunoreactivities as percentage area. Significance of differences between IBS and controls was determined by Mann–Whitney U test.

IBS, irritable bowel syndrome; PGP, protein gene product; SP, substance P; TRPV1, transient receptor potential vanilloid type-1.

When the IBS group was divided into symptom subgroups, there was no statistically significant difference in the numbers of TRPV1-immunoreactive fibres (IBS-D, n = 8, fibres/mm^2^, median 3.7, interquartile range 2.6–6.6; IBS-C, n = 7, 3.5, 2.1–4.9; and IBS-A, n = 8, 2.2, 2.0–5.2; ANOVA (analysis of variance) test, p = 0.23).

In order to exclude possible changes arising from differences in bowel preparation, the IBS group was also analysed by whether they had a flexible sigmoidoscopy (n = 7) or a colonoscopy (n = 16). TRPV1-immunoreactive fibres in the flexible sigmoidoscopy group (3.2; 2.1–4.5) and in the colonoscopy group (3.4; 2.2–5.6) were not significantly different (p = 0.6). Similarly PGP9.5-staining fibres (flexible sigmoidoscopy 5.0; 3.7–5.5 vs colonoscopy 5.0; 3.1–7.2; p = 0.8) and SP-staining fibres (flexible sigmoidoscopy 2.4; 1.2–2.8 vs colonoscopy 1.6; 0.7–3.4; p = 0.6) did not differ significantly between the two groups.

**Figure 2:**
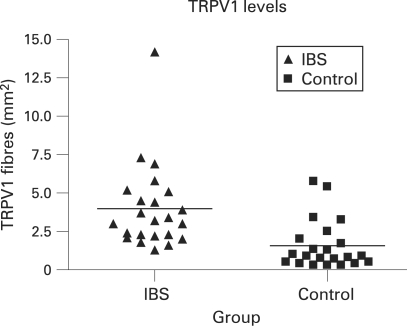
Transient receptor potential vanilloid type-1 (TRPV1) levels for the irritable bowel syndrome (IBS) and control groups; p<0.001.

As there was a difference in the age structure of the IBS and control groups, we also compared TRPV1 results in a subgroup of 13 subjects from the IBS and 13 subjects from the control group, both with an age range 50–75 years. In these age-matched subgroups, TRPV1-immunoreactive fibres were significantly increased in the IBS group (3.0; 2.2–4.8) compared with the control group (1.0; 0.6–2.1; p = 0.003). Multivariate linear regression analysis revealed that, unlike the presence of IBS, gender, age and type of bowel preparation/procedure were not significant independent predictors of TRPV1 levels (fig 2).

### Inflammatory markers

**Figure 3:**
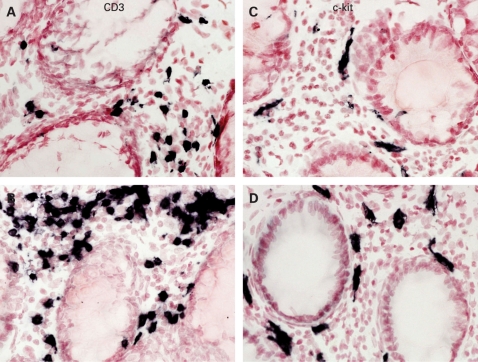
Photomicrographs showing CD3- and c-kit-immunoreactive staining cells in a control (A and C, respectively) and an irritable bowel syndrome rectosigmoid biopsy (B and D, respectively). Magnification ×40.

CD3+ T cells were seen scattered throughout the mucosa in all the specimens (fig 3A,B), including controls. There was a significantly greater percentage area of CD3+ cells in the IBS group compared with the controls (p = 0.03, table 4). Similar staining but of fewer cells was seen using the CD4 T cell antibody in both groups, and this was not statistically significant (p = 0.29, table 4). Mast cells immunoreactive to c-kit were also scattered throughout the mucosa (fig 3C,D), and were significantly elevated in the IBS group compared with controls (p = 0.02, table 4). With subgroup analysis of the flexible sigmoidoscopy versus colonoscopy group to exclude possible effects of differing bowel preparation, c-kit staining (flexible sigmoidoscopy 5.5; 3.4–6 vs colonoscopy 3.9; 2.6–4.5) did not differ significantly (p = 0.2) between the two groups.

### Pain severity, depression and anxiety

As expected, the median VASmax pain score was significantly higher in IBS patients than in controls (IBS 4.5; 1.4–6.5 vs control 0; 0–1.25; p = 0.002). Univariate linear regression analyses for all the immunoreactive species studied showed that only TRPV1 (p = 0.005, r = 0.43), c-kit (p = 0.004, r = 0.47) and age (p = 0.02; r = −0.34) data were statistically significantly related to the pain score VASmax. These three variables were introduced into a stepwise multivariate linear regression model which revealed that only TRPV1 (p = 0.005) and c-kit (p = 0.008) were significant predictors of VASmax.

**Figure 4:**
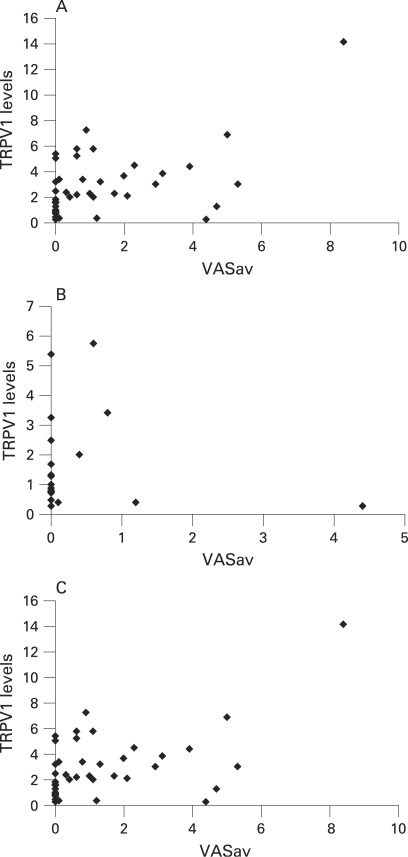
(A) Scatter plot to show the correlation between transient receptor potential vanilloid type-1 (TRPV1) levels and average pain scores (VASav) in irritable bowel syndrome (IBS) subjects. Pearson correlation r = 0.68, p<0.001. Spearman correlation r = 0.45, p = 0.02. (B) Scatter plot to show the correlation between TRPV1 levels and average pain scores (VASav) in control subjects. Pearson correlation r = −0.1, p = 0.34. (C) Scatter plot to show the correlation between TRPV1 levels and VASav in all subjects (controls and IBS). Pearson correlation r = 0.6, p<0.001. Spearman correlation r = 0.46; p<0.001.

The average VAS pain score was significantly greater in the IBS group; median IBS group 1.7; 0.6–4.1 vs control 0; 0–0.5 (p = 0.001). The average pain score also significantly correlated with TRPV1 in the IBS group (p = 0.02, r = 0.5, Spearman correlation) but not in the control group (p = 0.5; r = −0.02, Spearman correlation). If the single outlier in the IBS group with the high pain score is excluded and the data re-analysed to ensure that this point does not skew the p value, then there was still a positive correlation within the IBS group of pain scores with TRPV1 levels (Spearman correlation r = 0.4; p<0.05). When the two groups were combined together, the average pain score (VASav) also correlated with TRPV1 levels (p<0.001, r = 0.5 Spearman correlation) (see fig 4A–C). There was no correlation seen with pain score and CD3 or mast cell counts.

The age range of the IBS patients varied from 21 to 77 years, with six patients aged ⩽30 years. If these six patients aged ⩽30 years are selected as a subgroup, then there is still a positive correlation between TRPV1 expression and VASav (Spearman correlation r = 0.7, p = 0.04).

The HADS total scores in the IBS group (median 11.5, range 0–17) and control groups (median 9.5, range 2–25) were not statistically different (p>0.05). Anxiety subscale scores (IBS group median 7.5 vs control group median 6.5) and depression subscale scores (IBS group median 5 vs control group median 4) were also similar in the two groups. Similarly, BDI scores in the IBS group (median 9, range 1–24) did not significantly differ (p>0.05) from the control group (median 10.5, range 1–19).

## DISCUSSION

The demonstration of increased mucosal nerve fibres immunoreactive to TRPV1 in human colonic biopsies from IBS patients, and their correlation with the degree of abdominal pain, may provide a putative basis of IBS symptoms. The increased TRPV1 nerve fibres were seen throughout the IBS group, with no difference when the group was subclassified by Rome II definitions into IBS-D, IBS-C and IBS-A.

Winston *et al*^16^ have presented animal data which strengthen the role of TRPV1 in visceral hypersensitivity. Acetic acid colonic irrigation was used to induce a state of chronic visceral hypersensitivity in neonatal rats, and they found increased TRPV1 expression in dorsal root ganglia containing colonic afferent neurons. Treatment with a TRPV1 antagonist ameliorated sensitivity, when used both in the neonates prior to the acetic acid colonic irrigation, and in the adult hypersensitive rats. Their work suggests that TRPV1 is probably important both in initiating the process of visceral hypersensitivity and in maintaining it. In agreement with this, we have reported increased levels of TRPV1 in patients with idiopathic rectal hypersensitivity and faecal urgency: in this group of patients, the increased TRPV1 nerve fibres were correlated with rectal distension and heat thresholds (Chan *et al*^12^).

Our biopsies were obtained from IBS patients who were having either a colonoscopy or a flexible sigmoidoscopy. All control patients had a colonoscopy. We use different methods of bowel preparation in these two procedures; a phosphate enema for flexible sigmoidoscopy and Kleanprep for colonoscopy. There have been reports that bowel preparations may be responsible for histological changes^17^ with both enemas^18^ and colonoscopy sodium phosphate-containing bowel preparations.^19^ ^20^ However, despite the use of these two different preparations, all biopsies were reported histologically as normal so there were no apparent changes induced due the type of preparation used. Furthermore, the changes seen with immunohistochemical markers for immune cells were similar through the IBS group. Only seven of the 23 IBS patients underwent a flexible sigmoidoscopy, so the majority had a full colonoscopy with identical bowel preparation to the controls. There were no statistically significant differences in any staining markers between the patients who underwent a flexible sigmoidoscopy and those who had a colonoscopy in the IBS group.

Although our two groups differed slightly in age, multivariate regression analysis showed that this did not affect the results. Our IBS group had an age and sex spread similar to the IBS group published recently by Barbara *et al*^21^ investigating mast cell mediators in IBS. Furthermore, an age-matched subgroup analysis revealed that the TRPV1-immunoreactive nerve fibres were still significantly increased in the IBS group. The number of IBS patients and controls in the overall group and in this subgroup were powered at the 80% level to detect differences in TRPV1 fibre number of 0.5 and 1.5 fibres/mm^2^ at p<0.05.

Although both control and IBS biopsies were categorised as normal on standard histology and mucosal endoscopic views were normal, the CD3 count and c-kit expression were both significantly higher in the IBS group. Our findings are consistent with results from previous studies.^22^^–^24 These have reported increased mast cell numbers in colonic biopsies,^22^ ^23^ ^25^ as we have noted. Barbara *et al* found that mast cells which were proximal to nerves correlated with abdominal pain severity, suggesting a role for mast cells and their mediators in the altered sensorimotor pathophysiology of IBS.^22^ ^26^ Our results also showed that mast cell numbers/c-kit staining (but not mast cell tryptase staining—data not shown) correlated positively with abdominal pain scores. Dong *et al*^27^ took biopsies from patients with IBS from different ileocolonic sites during colonoscopy and, using immunohistochemistry techniques, demonstrated an increase in mast cell numbers and SP-staining immunoreactive fibers, and also a spatial correlation between the two. Increased T lymphocytes have been reported in both infectious and non-postinfectious IBS.^28^^–^30 These results implicate that a low-grade inflammatory process in IBS could be involved in the pathogenesis of IBS, and that mast cell interaction with nerve fibres including SP-positive fibres may be important, in accord with our findings. From 6% to 17% of unselected IBS patients cite an infectious trigger for their onset of IBS symptoms, and prospective studies suggest an incidence of postinfectious IBS which follows a bacterial gastroenteritis of 4–31%.^31^ Other inflammatory cells have also been linked to postinfectious IBS; Dunlop *et al* reported 20% increased numbers of enterochromaffin (EC) cells^30^ which contain serotonin 3 months after the initial infective gastroenteritis when compared with controls. When EC cells are triggered, they release serotonin which acts on nearby receptors and nerve endings. Furthermore there have been reports of increased cytokines in peripheral blood of IBS patients.^32^ ^33^ These findings strengthen the role of an inflammatory process in triggering IBS. Although there is increasing evidence that sensorimotor dysfunction in IBS is likely to result from an interaction between mucosal immune cell mediators, nerve fibres and muscle layers, further studies into the triggering and predisposing mechanisms are needed. Various factors proposed include infective gastroenteritis, genetic causes, food allergies and alterations in gut microflora.^34^ Interestingly, TRPV1 expression has been reported on mast cells.^35^ Stander *et al*^35^ reported VR1 expression on dermal mast cells, suggesting a role in activation of these cells and perpetuation of inflammation.

TRPV1 is likely to be activated by the products of inflammation in IBS, and, through its upregulation, may contribute to symptoms including pain. There was evidence of nerve fibre sprouting in IBS, as PGP9.5 nerve fibres were increased. Inflammation-mediated upregulation of TRPV1 is well established, and has been shown to involve various mechanisms including nerve growth factor (NGF) and p38MAP kinase, along with sensitisation of TRPV1 by bradykinin B2 via intracellular enzymatic pathways. NGF production in peripheral tissues is enhanced by inflammation, and NGF is taken up and transported in a retrograde manner by nerve fibres to their cell bodies, leading to nerve sprouting and increased expression of TRPV1 and SP.^36^ Not only does NGF sensitise TRPV1 receptors to protons, enhancing their effect, but it also increases expression of TRPV1. Increased NGF, and recently trk A, expression has been reported in acute inflammatory bowel disease.^37^ ^38^ Ji *et al*^38^ have shown that the increase in TRPV1 levels which occurs 12–24 h after inflammation is by an NGF-mediated p38 kinase pathway. TRPV1 activity is modulated by inflammatory mediators including bradykinin and prostaglandins, probably by cAMP-dependent protein kinase (PKA)- or protein kinase C (PKC)-mediated phosphorylation of the receptor.^39^ Generally, protein kinase-mediated phoshorylation of the TRPV1 receptor results in sensitisation, and dephosphorylation by protein phosphatases results in desensitisation.^40^ NGF immunostaining has been difficult to obtain in gut specimens, especially mucosal biopsies as we have used here. Future studies would be useful to assay NGF, with different processing of tissues.

Our findings, of increased total nerve fibres, and nerve fibres immunoreactive to TRPV1 and SP in IBS, may thus all be mediated via the effects of NGF. There are other mechanisms, however, that may also modulate TRPV1 function—Sugiura *et al*^41^ reported data from a mouse model suggesting that 5-hydroxytryptamine (5-HT, serotonin) receptor activation may enhance the responsiveness of the TRPV1 receptor to acid and temperature, and thereby contribute to peripheral sensitisation. It is thought that this response is mediated via 5-HT_2_ or 5-HT_4_ receptors. Serotonin signalling alterations seen in IBS may act in part via TRPV1 sensitisation.

TRPV1 activation produces an influx of calcium and sodium ions, along with release of neuropeptides (SP, CGRP). This in turn triggers and promotes the process of neurogenic inflammation. The oral TRPV1 antagonist JNJ 10185734 has been reported to attenuate dextran sulfate sodium (DSS)-induced colitis in mice, suggesting a possible role for TRPV1 in initiation or maintenance of inflammation in the gut.^42^ Other previous animal studies strengthen the view that TRPV1 is involved in inflammation and hyperalgesia. In a rat model of DSS colitis,^43^ neonatal animals chemically denervated of TRPV1 fibres by treatment with capsaicin, and those given a TRPV1 antagonist in established DSS colitis, were both protected from the damaging effects of DSS.

Co-localisation studies would give further information as to the origin of the nerve fibres under study, subject to the availability of good antibodies for TRPV1 and SP raised in different species. Such studies may also provide insight into the relationship of these markers to abdominal pain score. The two primary antibodies used in this study are both anti-rabbit (table 4), thereby precluding co-localisation by immunohistochemistry.

There have been reports of a higher incidence of anxiety and depressive symptoms in IBS patients than in controls,^44^ ^45^ but our IBS patients had similar BDI and HADS scores to those of the control group. Central factors are likely to play an important role in the generation of pain and visceral hypersensitivity as well as peripheral mechanisms.^34^ In this study we focused on the peripheral sensitising mechanisms involving low-grade inflammation and neuronal interactions in the generation of pain.

In summary, we present evidence that TRPV1 nerve fibres are increased in the mucosa of IBS patients. Our results provide a mechanism which may contribute to the pathophysiology of pain in IBS, and clinical trials with TRPV1 antagonists are warranted in this condition.
